# Case report: Neglected subacute thyroiditis: a case following COVID-19 vaccination

**DOI:** 10.3389/fmed.2024.1349615

**Published:** 2024-03-08

**Authors:** Shuai Yang, Ting Guan, HuanYi Yang, YiRong Hu, Yan Zhao

**Affiliations:** ^1^School of Sports Medicine and Health, Chengdu Sport University, Chengdu, China; ^2^Department of Endocrinology and Metabolism, West Chengdu Hospital, Chengdu, China

**Keywords:** COVID-19, subacute thyroiditis, SARS-CoV-2 vaccine, autoimmunity, fever

## Abstract

We report a case of overlooked Subacute Thyroiditis (SAT) potentially induced by the administration of a COVID-19 vaccine. This case prompted a thorough review of the existing literature to elucidate possible mechanisms by which immune responses to the COVID-19 vaccine might precipitate thyroid damage. The primary objective is to enhance the clinical understanding and awareness of SAT among healthcare professionals. Subacute thyroiditis is a prevalent form of self-limiting thyroid disorder characterized by fever, neck pain or tenderness, and palpitations subsequent to viral infection. The development of numerous SARS-CoV-2 vaccines during the COVID-19 pandemic was intended to mitigate the spread of the virus. Nevertheless, there have been documented instances of adverse reactions arising from SARS-CoV-2 vaccines, such as the infrequent occurrence of subacute thyroiditis. While the majority of medical practitioners can discern classic subacute thyroiditis, not all cases exhibit typical characteristics, and not all systematic treatments yield positive responses. In this study, we present a rare case of subacute thyroiditis linked to the administration of the SARS-CoV-2 vaccine. A previously healthy middle-aged female developed fever and sore throat 72 h post-inoculation with the inactivated SARS-CoV-2 vaccine. Initially attributing these symptoms to a common cold, she self-administered ibuprofen, which normalized her body temperature but failed to alleviate persistent sore throat. Suspecting a laryngopharyngeal disorder, she sought treatment from an otolaryngologist. However, the pain persisted, accompanied by intermittent fever over several days. After an endocrinology consultation, despite the absence of typical neck pain, her examination revealed abnormal thyroid function, normal thyroid antibodies, heterogeneous echogenicity on thyroid ultrasonography, and elevated levels of Erythrocyte Sedimentation Rate (ESR) and C-Reactive Protein (CRP). These findings led to a consideration of the diagnosis of SAT. Initially, she was treated with non-steroidal anti-inflammatory drugs (NSAIDs) for her fever, which proved effective, but her neck pain remained uncontrolled. This suggested a poor response to NSAIDs. Consequently, steroid therapy was initiated, after which her symptoms of fever and neck pain rapidly resolved.

## Introduction

Subacute thyroiditis, also known as granulomatous thyroiditis or De Quervain’s thyroiditis, represents a relatively rare yet significant thyroid disorder characterized by its self-limiting nature (1). It leads to thyroid destruction, resulting in thyrotoxicosis, which can exacerbate comorbid conditions such as respiratory distress and diabetes, and in severe cases, lead to multi-organ failure and potentially evolve into permanent hypothyroidism (2). Patients typically present with classic symptoms of upper respiratory tract infection, fever, anterior neck pain, and thyroid dysfunction. Despite decades of research, the pathogenesis and critical factors influencing the clinical course of this disease remain incompletely understood. Reports of SAT following COVID-19 vaccination are scarce; however, there have been instances of SAT following other vaccinations, including influenza and hepatitis B vaccines (3, 4).

Since the onset of the COVID-19 pandemic in 2020, an unparalleled global public health emergency has been instigated. In light of this crisis, scientists, medical professionals, and researchers worldwide have been diligently engaged in the pursuit of an efficacious vaccine against this rampant infectious ailment. Over time, numerous COVID-19 vaccines have been triumphantly formulated, prompting governments across the globe to actively initiate large-scale vaccination campaigns, thereby fostering renewed optimism in curtailing the epidemic. During the process of vaccination, adverse reactions induced by vaccines are a matter of significant apprehension, necessitating continuous surveillance and comprehensive investigation (5). Among these reactions, the occurrence of subacute thyroiditis subsequent to vaccination has sparked extensive deliberation and thorough exploration into the possible correlation between vaccines and thyroid disease.

## Case presentation

**Figure 1:**
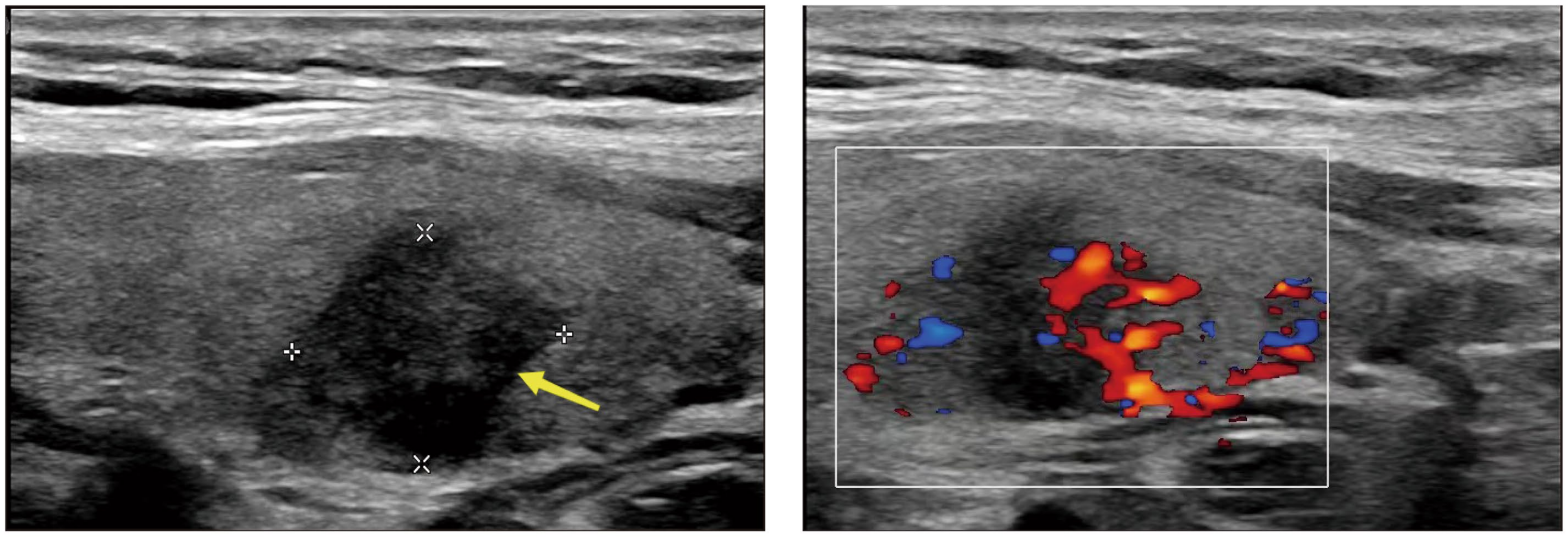
The echo of thyroid is decreased, and the density is uneven.

The patient is a middle-aged woman who has not received any other vaccines within the past six months and has no history of COVID-19 infection. There was no history of thyroid disease diagnosis in the patient’s immediate family, encompassing both parents and offspring. After receiving the first dose of the SARS inactivated vaccine (BBIBP-CorV), she developed a sore throat and fever 72 h later. Her body temperature reached up to 38.6°C and she did not experience coughing, sputum production, palpitations, or dyspnea. She self-administered cephalosporin antibiotics and ibuprofen, but her pain symptoms did not significantly improve. An examination at the otolaryngology department confirmed that her tonsils were enlarged at I°, the posterior pharyngeal and lateral pharyngeal walls were congested and swollen, and the thyroid gland was mildly tender but not significantly enlarged. Other tests, including COVID-19 nucleic acid, antibodies, influenza A and B viruses, and respiratory pathogens, were negative. Nevertheless, it was observed that the WBC and neutrophil ratios exhibited a slight increase, whereas the levels of CRP and ESR were significantly elevated and displayed a strong correlation with SAT. Her liver and kidney function were normal, and there were no obvious abnormalities found in abdominal color ultrasound, chest CT, and electrocardiogram. She received intravenous infusion of cefazolin sodium, vitamin C, and nebulization of the throat as treatment. During the treatment, she experienced recurrent fever and repeated COVID-19 nucleic acid tests were negative. After consultation with the endocrinology department, it was discovered that her thyroid-stimulating hormone (TSH) was decreased, while free thyroxine (FT4), free triiodothyronine (FT3), and thyroglobulin (Tg) were increased. Thyroglobulin antibody (TgAb) and anti-thyroid peroxidase antibody (TPOAb) were not found to be special (Table 1). Thyroid ultrasound revealed hypoechoic areas with blurred edges, irregular shapes, and decreased blood vessels, indicating thyroiditis and cysts in the right lobe of the thyroid gland (TI--RADS class 2) (Figure 1). The patient independently administered ibuprofen prior to hospitalization. Although her body temperature briefly improved, her symptoms of a sore throat persisted, and she encountered recurrent fevers and a sore throat throughout her hospitalization. After excluding other infections and potential sources of fever and sore throat, subacute thyroiditis was contemplated based on the patient’s medical history and examination findings. Once the diagnosis was verified, it was established that the ibuprofen consumed prior to admission did not alleviate the symptoms of a sore throat. The therapeutic efficacy of non-steroidal drugs was deemed inadequate for the patient, thus prompting the administration of prednisone 10 mg qd. Following the administration of prednisone, the patient experienced alleviation of neck pain symptoms, normalization of body temperature, and absence of subsequent recurrence during the subsequent outpatient follow-up.

**Table 1:** Laboratory investigations.

Variable	Reference range adult	Initial visit	Follow-up		
			2 weeks	4 weeks	2 months
TSH	0.28–4.12 nmol/L	0.02	1.19	3.23	3.38
TT3	1.20–3.15 nmol/L	3.06			
TT4	61.13–164.74 nmol/L	174.06			
FT3	3.05–6.85 pmol/L	11.61	6.72	5.53	5.24
FT4	12.0–21.50 pmol/L	37.43	20.46	17.32	17.79
Anti-TPO	<9 IU/mL	9.0			7.4
Anti-TG	<4 IU/mL	0.1			0.1
Tg	1.15–130.77 ng/mL	266.96			103.29
ESR	<20 mm/h	108	13		3
CRP	<5.0 mg/L	62.43	5.13		1.42
WBC	4.0–10.0 × 10^9^/mL	11.63	7.48		3.25

WBC, white bood cell; CRP, c-reactive protein; ESR, erythrocyte sedimentation rate; TSH, thyroid stimulating hormone; TT3, total Triiodothyronin; TT4, total thyroxine; FT3, tree triiodothyronine; FT4, tree thyroxine; Anti-TPO, anti-thyroid teroxidase ntibody; Anti-TG, anti-thyroglobulin; Tg, thyroglobulin.

## Discussion

Subacute thyroiditis is a thyroid disorder that typically manifests 2–8 weeks following viral infection and exhibits a higher prevalence among women, particularly within the 40–50 age bracket (6). The classic initial clinical manifestations of SAT include neck pain or tenderness, fever, palpitations, accompanied by a range of thyroid dysfunction indicators, with early-stage thyrotoxicosis as the primary clinical feature (7). These symptoms result from a regressive process affecting the thyroid follicular epithelium, leading to the release of thyroglobulin, thyroxine, and an assortment of other iodine-rich molecular fragments into the systemic circulation, thus indicating a potential trend towards hypothyroidism in this patient subgroup (1, 7, 8). The confirmation of SAT diagnosis depends on a synergistic assessment encompassing clinical symptomatology, an array of laboratory diagnostic techniques, and radiological imaging findings. Typical laboratory markers comprise thyroid functional abnormalities, identifiable thyroid lesions via ultrasound, and increased titers of serum thyroid antibodies, with the majority of patients testing positive for thyroid peroxidase (TPO) antibodies and a minority for thyroglobulin antibodies. The diagnostic process for SAT invariably involves systematically ruling out other thyroid pathologies, including hyperthyroidism and subclinical hyperthyroid variants. The etiology of SAT is inherently multifaceted, encompassing factors such as viral invasion of the thyroid, unique immune response mechanisms, and genetic predispositions (8–10).

The primary approach to managing subacute thyroiditis involves alleviating clinical symptoms and addressing potential complications. Patients frequently necessitate sufficient rest, analgesics, and nonsteroidal anti-inflammatory drugs to mitigate pain and elevated body temperature. In severe cases, oral corticosteroids may be administered to alleviate symptoms of thyroiditis, if deemed necessary (11, 12). The majority of patients are anticipated to experience recovery within a span of weeks to months, although a subset of individuals may experience a delay in the onset of chronic thyroiditis (11).

In light of the emergence of the novel coronavirus, various SARS-CoV-2 vaccines have been formulated, encompassing mRNA vaccines (Pfizer/BioNTech, Moderna), viral vector-based vaccines (ChAdOx1 nCoV-19 vaccine, CanSino Biologics, Gamaleya Institute, Johnson & Johnson), inactivated vaccines (CoronaVac, Sinovac, Bharat Biotech BBV 152, Sinopharm BIBP), and protein subunit-based vaccines (Novax, Chinese Academy of Sciences) (13–17).

Globally, there have been reported incidents of adverse reactions from diverse vaccine types, but on the whole, instances of subacute thyroiditis ensuing from vaccination are uncommon (17–20). Such occurrences frequently manifest within a few days to weeks post-vaccination and exhibit characteristic symptoms of thyroiditis, encompassing severe neck pain, thyroid swelling, and thyroid function disruption (15). These reports meticulously detail patients presenting with thyroiditis symptoms post-vaccination, dismiss other influencing factors, and provide comprehensive information concerning their clinical manifestations, laboratory examination outcomes, and additional related details. The objective of these reports is to elucidate potential correlations between vaccination and thyroid disorders and alert potential risks. Postpondered from case reports (5, 17, 19), SARSCoV-2 vaccination can induce 70% of mRNA-inducing individuals vaccinated, with viral vector-based vaccines accounting for 18% of reported cases. Conversely, SARSCoV-2 inactivated vaccines infrequently evoke SAT. An analysis of relevant case data indicates that 55% of patients experienced SAT following their first dose of SARS-CoV-2 vaccine, while this proportion rises to 44% after the second dose (5, 6, 17). Concerning the duration between administration of the vaccine and symptom appearance, a median time of 10 days was identified, ranging from as short as 12 h to as long as 84 days, which varies from the typical presentation of SAT (15, 20).

The precise etiology of subacute thyroiditis following vaccination remains uncertain; however, several studies propose that vaccines may elicit immune system activation, particularly the autoimmune response, resulting in potential damage to thyroid tissue (10). This observation implies that vaccine-related thyroid complications may pose a rare yet tangible hazard, particularly among individuals with pre-existing thyroid conditions or genetic susceptibility (21–25).

Autoimmunity has been explained by several hypotheses, including molecular mimicry (26, 27). Some scholars are positing that the immune response triggered by the SARS-CoV-2 spike protein and the SARS-CoV-2 nucleoprotein results in the generation of cross-reactive antibodies (23, 26, 28). These antibodies, in turn, engage with various tissue antigens, including thyroid tissue, thereby instigating the development of autoimmune diseases, such as SAT (29, 30). The mechanism is SARS-CoV-2 single-stranded RNA viruses with similar structures to the novel coronavirus, and different types of COVID19 vaccines also share a common feature, namely molecular mimicry between S protein, viral protein, and human tissue, and the immune response to SARSCoV2 spike protein and Nucleo protein leads to cross-reactivity to produce antibodies, and their interaction with different tissue antigens, including thyroid tissue, leads to autoimmune thyroid disease (25–32). Simultaneously, the S protein’s interaction with the angiotensin-converting enzyme 2 (ACE2) receptor situated on the cellular membrane facilitates the virus’s adherence to said receptor, subsequently initiating viral entry into the cell and consequent infection (28, 33, 34).

Furthermore, adjuvants have been found to augment the immunogenicity of vaccines, bolster innate and autoimmune responses, and potentially trigger the production of autoantibodies or local/systemic inflammation (35–40). Nevertheless, when adjuvants are present, viruses have the capability to elicit diverse inflammatory and autoimmune reactions in genetically vulnerable populations while interacting with host cells (37, 40–43). COVID-19 vaccines incorporate various excipients, including aluminum hydroxide or aluminum salts (found in the Coronavac vaccine), polysorbate 80 (used in the AstraZeneca vaccine), and polyethylene glycol (PEG) lipid conjugates, stable lipid nanoparticles, among others (29, 36). These excipients may serve as adjuvants in mRNA vaccines (such as Pfizer/BioNTech) and water–oil emulsion formulations, potentially leading to autoimmune or allergic responses following COVID-19 vaccination (27, 44, 45).

Moreover, research has demonstrated that metabolites derived from SARS-CoV-2 disrupt the configuration and operation of human leukocyte antigen (HLA), a phenomenon associated with the resemblance between human leukocyte antigen (HLA) genes and SARS-CoV-2 antigens (43, 46–49). This resemblance renders certain individuals more prone to thyroid disease (47, 50). Simultaneously, specific variants of HLA (such as HLAB35) have exhibited heightened susceptibility to this virus-susceptible antigen (43, 48). The activation of the HLAB35 antigen complex has the potential to initiate immune-mediated damage to thyroid follicular cells (5, 51). Additionally, there is a noteworthy concern regarding certain factors previously identified as risk factors (such as individual or familial autoimmune disease or pregnancy) or predictors (such as smoking, high-pressure environment, or drug intake), as they can influence the development of autoimmunity following COVID-19 vaccination (22, 49). In many instances, this can result in the manifestation of inflammatory thyroid disease.

According to the inflammatory factor storm theory, subacute thyroiditis caused by most SARS-CoV-2 vaccines typically presents with inflammatory symptoms such as neck pain, myalgia, and fever. Additionally, thyroid color ultrasound examination may reveal structural abnormalities and hypoechoic areas in the thyroid, and some patients may experience thyroid enlargement. Blood biochemical tests have shown that almost all patients exhibit thyrotoxicosis and elevated levels of serum inflammatory markers like CRP/ESR. Most patients respond well to treatment with NSAIDs or prednisolone (or other steroids), indicating an inflammatory response. The mechanism behind this inflammatory response may be associated with the release of cytokines, which can initiate a chain reaction resulting in increased levels of circulating interleukin-6 (IL-6), interleukin-1 receptor antagonist (IL-1RA), chemokine 2 (CCL2), chemokine 8 (CCL8), as well as chemical antagonists of T cells or natural killer (NK) cells including chemoattractant 9 (CXCL9) and chemokine 16 (CXCL16) (24, 38, 39, 52).

However, due to the frequent overlap in clinical manifestations between SAT and other diseases, there is a potential for misdiagnosis or missed diagnosis. Consequently, clinicians must be highly vigilant when encountering patients who exhibit thyroid-related symptoms post-vaccination, particularly in cases following COVID-19 vaccination. If vaccine-induced SAT is suspected, the priority should be to conduct timely and comprehensive clinical assessments along with thyroid function tests, to lay a solid foundation for accurate diagnosis and the prompt implementation of an appropriate treatment plan. Additionally, healthcare professionals need to be aware of the potential link between vaccinations and thyroid issues in order to provide more precise medical guidance.

## Conclusion

This article aims to explore the possible relationship between vaccines and subacute thyroiditis by examining a case of misdiagnosis due to overlooked SAT, as well as reviewing recent case reports from various countries. It suggests that vaccines may trigger an immune response leading to the development or exacerbation of SAT, especially in females and individuals with existing thyroid disorders or genetic predispositions. However, the incidence of SAT associated with COVID-19 vaccines appears to be relatively low, which may be due to challenges in accurate diagnosis as typical clinical symptoms are often overlooked or masked and mistaken for other conditions, and are mostly transient in nature. Conclusive assessment can be aided by certain biochemical tests, such as elevated serum inflammatory markers and thyrotoxicosis. Ultrasound examination may reveal thyroid enlargement, structural changes, and hypoechoic areas, while reduced blood flow can be observed through thyroid Doppler imaging. Treatment typically involves the use of non-steroidal anti-inflammatory drugs or corticosteroids. Notably, the likelihood of SAT induced by inactivated SARS-CoV-2 vaccines during the COVID-19 pandemic seems lower. Patients with fever and neck pain may incorrectly use non-steroidal anti-inflammatory drugs, potentially affecting the presentation of temperature and neck pain. Moreover, relying solely on symptomatic treatment with oral medications may mask the progression of the disease. Although most mild cases resolve spontaneously, some individuals may experience delayed progression, leading to hypothyroidism, thyroid storm, or other serious complications, potentially exacerbating the condition or posing a life-threatening risk. Therefore, medical experts must remain vigilant when assessing individuals with thyroid symptoms, particularly following COVID-19 vaccination, to ensure accurate diagnosis and timely treatment, as early clinical and thyroid function assessments are crucial for confirming diagnosis and expediting treatment. Vaccination is a critical public health intervention that significantly combats the spread of novel coronavirus and mitigates the severity of the pandemic. Thus, vaccination programs should closely monitor and document any adverse reactions related to thyroid complications to fully understand their incidence and characteristics. Future research should further investigate the association between vaccination and thyroid disorders to strengthen the scientific basis for formulating immunization strategies that prioritize public health and welfare.

## Data availability statement

The original contributions presented in the study are included in the article/Supplementary material, further inquiries can be directed to the corresponding author.

## Ethics statement

Written informed consent was obtained from the individual(s) for the publication of any potentially identifiable images or data included in this article.

## Author contributions

SY: Writing – original draft. TG: Data curation, Writing – review & editing. HY: Formal analysis, Writing – review & editing. YH: Conceptualization, Supervision, Writing – review & editing. YZ: Conceptualization, Project administration, Supervision, Writing – review & editing.
